# Development and validation of the health literacy environment scale for Chinese hospitals from patients’ perspective

**DOI:** 10.3389/fpubh.2023.1130628

**Published:** 2023-06-02

**Authors:** Yingge Tong, Yixue Wu, Zhiqing Han, Zihao Xue, Yeling Wei, Shanyuan Lai, Ziyi Chen, Miaoling Wang, Siyi Chen

**Affiliations:** ^1^School of Nursing, Hangzhou Normal University, Hangzhou, China; ^2^Department of Operating Room, Affiliated Sir Run Run Shaw Hospital, Zhejiang University School of Medicine, Hangzhou, China

**Keywords:** health literacy, environment, scale, hospital, person-centered care

## Abstract

**Introduction:**

While the research on improving individual health literacy by promoting individual skills and abilities is increasing, less attention has been paid to the complexities of the healthcare environment that may influence patients’ ability to access, understand, and apply health information and health services to make health decisions. This study aimed to develop and validate a Health Literacy Environment Scale (HLES) that is suitable for Chinese culture.

**Methods:**

This study was conducted in two phases. First, using the Person-Centered Care (PCC) framework as a theoretical framework, initial items were developed by using the existing health literacy environment (HLE) related measurement tools, literature review, qualitative interviews, and the researcher’s clinical experience. Second, scale development was based on two rounds of Delphi expert consultation and a pre-test conducted with 20 hospitalized patients. Using 697 hospitalized patients from three sample hospitals, the initial scale was developed after item screening and its reliability and validity were evaluated.

**Results:**

The HLES comprised 30 items classified into three dimensions as follows: interpersonal (11 items), clinical (9 items), and structural (10 items) dimensions. The Cronbach’s α coefficient of the HLES was 0.960 and the intra-class correlation coefficient was 0.844. The confirmatory factor analysis verified the three-factor model after allowing for the correlation of five pairs of error terms. The goodness-of-fit indices signified a good fit for the model (*χ*^2^/df = 2.766, RMSEA = 0.069, RMR = 0.053, CFI = 0.902, IFI = 0.903, TLI = 0.893, GFI = 0.826, PNFI = 0.781, PCFI = 0.823, PGFI = 0.705). The item-content validity index ranged from 0.91 to 1.00, and the scale-content validity index was 0.90.

**Conclusion:**

The HLES had good reliability and validity and provides a patient perspective tool for evaluating HLE and a new perspective for improving health literacy in China. That is, healthcare organizations make it easier for patients to access, understand, and use health information and service. Further studies about the validity and reliability of HLE should include other districts and different tiers or types of healthcare organizations.

## 1. Introduction

Individual health literacy is “the degree to which individuals have the capacity to obtain, process, and understand basic health information and services needed to make appropriate health decisions” (1). Inadequate health literacy has become a significant global public health problem, although data on this are still unavailable. For instance, only 12% of American adults had proficient health literacy in 2003, according to National Center for Education Statistics (2). Similarly, a survey of the European Health Literacy Project (HLS-EU) revealed that 12.4% of participants had insufficient health literacy and 47.6% of participants had limited health literacy (3). Health literacy directly affects an individual’s or community’s health awareness and determines the choice of health decisions and actions. Individual health literacy correlates with social determinants of health, such as education, income, and environment, to influence health (4). However, individual health literacy changes dynamically. Even if individuals have adequate health literacy, it would be difficult for them to access, understand, and apply health information and health services to make health decisions in an unfamiliar environment and a jargon-filled doctor-patient communication process (5).

Most measures to improve health literacy are designed to enhance individuals’ skills and abilities (6–8). However, recent studies (9–11) have shown that health literacy results from the balance between individual skills and abilities, and its demand has intensified owing to the complexities of healthcare systems (12, 13). Therefore, it is critical to make healthcare organizations “easier for people to navigate, understand, and use information and services to take care of their health” (5). The concept of health literacy environment (HLE) has been developed to leverage the role of healthcare organizations and healthcare professionals in promoting health literacy, to reduce the complexity of the healthcare environment and health inequities. According to Rudd et al. (14), HLE is “a healthcare facility that represents the expectations, preferences, and skills of those providing health information and services.” In 2014, the Australian Commission on Safety and Quality in Health Care (ACSQHC) crafted two definitions for health literacy: individual health literacy and HLE. It defined HLE as the infrastructure, policies, processes, materials, people, and relationships that make up the health system and have an impact on the way people access, understand, appraise, and apply health-related information and services (15). Since the HLE concept was proposed in 2006, a growing number of agencies, institutions, and scholars have developed similar concepts, focusing on the role of healthcare organizations in promoting health literacy. Trezona et al. (16) defined Organizational Health Literacy Responsiveness (Org-HLR) as a healthcare organization’s responsiveness to handle health literacy promotion through the system-level way to promote equitable access and engagement, meet the health literacy needs of people and the community, and support people to engage in decisions about their health. The *Healthy People 2030,* which was initiated by the United States Department of Health and Human Services (HHS), defined organizational health literacy (OHL) as “the degree to which organizations equitably enable individuals to find, understand, and use information and services to inform health-related decisions and actions for themselves and other” (17). Although these concepts were developed in different contexts, they all focus on reducing the complexity of healthcare organizations to make it easier for people to access, understand and use health information and health service.

Assessment shines a light on the barriers and enablers that may be impacting the quality of service (18). Identification of burdensome health literacy demands can be the first step in reducing barriers and providing more accessible and effective care (19). Through the literature review, we found that there is only one tool to measure HLE (14, 20). In 2006, Rudd and Anderson (14) developed the Health Literacy Environment of Hospitals and Health Centers (HLEHHC), the first HLE measurement tool in America, to assist administrators and medical workers in considering the HLE of their healthcare facilities and analyzing ways to reduce demands, to better serve their patients. The HLEHHC has five sections (Navigation; Print communication; Oral exchange; Technology; Policies and protocols) and 100 criteria. It is scored by the hospital’s administrators, medical workers, and internal quality managers in a checklist format (1 = This is something that is not done; 2 = This is done but needs some improvements; 3 = This is done well). The total score is 300. A score ranging between 0–100, 101–200, and 201–300 represents “Begin a focused initiative to eliminate literacy-related barriers,” “Augment efforts to eliminate literacy-related barriers,” and “Continue to monitor and eliminate literacy-related barriers,” respectively. In 2019, Rudd and Anderson (20) revised and updated the HLEHHC to create the HLEHHC version 2 (HLE2). The HLE2 is organized into five sections, and 135 criteria: (1) Organizational policies, (2) Institutional practices, (3) Navigation, (4) Culture and language, and (5) Communication: print materials, forms, websites, and patient portals. HLE2 is scored by the hospital’s internal quality managers in a checklist format and patients in a 5-likert scale for the dimension of navigation. If the score is below 50%, it implies that actions are needed to begin a health literacy initiative to eliminate literacy-related barriers in this area, and if the score ranges from 86 to 99%, it represents the need to continue monitoring, to consider a study comparing baseline values with values at a later date, and to share experiences and findings with others. Although the HLEHHC and HLE2 have not been tested for reliability and validity, the former has been widely used in the Occident. In 2010, Smith et al. (21) used the HLEHHC to evaluate a rehabilitation center and a senior independent living facility and found that the measures in the Navigation, Print communication, and Oral exchange sections needed urgent improvement. In 2016, Oelschlegel et al. (22) conducted a systematic assessment over 6 months in a medical center in the United States. By analyzing 150 print patient education documents, interviewing nearly 300 patients, receiving feedback from 7 navigators, and measuring 77 administrators’ knowledge of policies and protocols, the score obtained by the center was 218.57, suggesting that the it should continue to monitor and eliminate literacy-related barriers, among which the sections “Oral exchange” and “Policies and protocols” needed to be further improved. In 2017, Palumbo et al. (23) revised the HLEHHC and translated it into Italian. The study included three large public hospitals in Italy. The scores of the sections “Technology” and “Policies and Protocols” were low, and thus countermeasures were put forward to meet the special needs of people with low health literacy through the introduction of specific technologies and the settlement of policies and tailored protocols. Both the HLEHHC and the HLE2 have detailed and comprehensive evaluation content and mainly objective indicators. However, Unlike the HLEHHC, the HLE2 takes into account the importance of patient experience, and its invites patients to participate in the evaluation of the navigation which was not the entirety of HLE. As healthcare systems become increasingly complex, patients may encounter difficulties finding and receiving health care services, using materials, navigation, filling forms, and offering consent for procedures (24–26). Evaluating HLE from the patient’s perspective can directly and truly acknowledge the deficiencies of the hospital that need to be improved or the experience worth promoting in the process of assisting patients to obtain, understand, and use health information and service (19). Therefore, there is an urgent need to develop a HLE scale based on the perspective of patients.

The National Health Commission of the People’s Republic of China reported that the health literacy rate in China is 25.40%, which means that only about one in four people in China has acquired basic knowledge and skills in health (27). Studies have also revealed that low health literacy is associated with poor health outcomes, including less frequent screening for diseases, high rates of disease and mortality, increased hospitalization rates, and medical costs (28, 29). To address the challenges of low health literacy, China’s government and health administration departments have implemented a policy to improve health literacy and require healthcare organizations to assume corresponding responsibilities. In May 2014, the National Health Commission of the People’s Republic of China initiated (30) the “*National Plan of Health Literacy Promotion Initiatives (2014–2020).*” This document recommended an establishment of a long-term and working mechanism that features leadership by the government, multi-departmental cooperation, and whole-society participation, and brings essential medical literacy promotion into the comprehensive assessment of healthcare organizations as well as the Health Promoting Hospital Initiative.

In 2019, the State Council, China’s Cabinet, launched “*Actions to Build a Healthy China (2019–2030).*” This government document is an overall design and strategy for promoting the health of all people for more than 10 years. The *Healthy China Initiative* takes health literacy promotion as the prerequisite for the health of all people. At the same time, it requests healthcare professionals to advise health matters initiatively and to establish the performance appraisal mechanism for health promotion and education conducted by medical and healthcare organizations and professionals (31). It is the first time the State Council has requested healthcare organizations to promote health literacy.

In contrast to government documents, most healthcare organizations focus on treating disease rather than promoting health literacy. Few practices are related to HLE in Chinese healthcare organizations. Only one public hospital and one community healthcare center are engaged in health literacy promotion (32, 33). The former carries out health literacy promotion practices in four aspects: (1) developing organizational rules and regulations, (2) forming a health popular science team, (3) setting up a health popular science platform, and (4) innovating the form of health popular science activities. The latter practices health promotion by: (1) establishing a leading group of health literacy promotion, (2) improving management mechanisms, and (3) conducting staff training, evaluating action, and targeting health literacy promotion activities. The two healthcare organizations focused on improving the accessibility and radiation of health popular science knowledge. However, their practices should have included other HLE-related areas, such as reducing services’ complexity and improving information comprehension.

Since the *Actions to Build a Healthy China (2019–2030)* was first published, the Chinese government has been releasing a document each year (from 2020 to 2022), setting out the annual work points of the *Healthy China Initiative*. In 2020, the government established “the performance appraisal mechanism for health promotion and education conducted by medical and healthcare organizations and professionals” as one of the high-priority annual tasks (34). The following year, 2021, the government endorsed this task as a key one to accelerate the implementation of the *Healthy China Initiative* (35). In 2022, the government continued the promotion of this task (36). This showed that the government attached great importance to the assessment of healthcare organizations for advancing health literacy promotion.

However, no government regulations or evaluation tools are currently available to assess the performance of health education and health promotion conducted by healthcare organizations and professionals in China. Only one study by Tong et al. (37) translated Kowalski et al.’s (38) health literate healthcare organization 10-item questionnaire (HLHO-10) into Chinese. The Chinese version of HLHO-10 (HLHO-10-C) had adequate reliability and validity. The HLHO-10-C was used to investigate 24 healthcare organizations in China and the results revealed that healthcare organizations had the highest scores for item 6 (communication standards) and item 9 (high risk) and the lowest scores for item 3 (workforce) and item 4 (inclusion of the served).

However, the HLHO-10 was developed in the context of Western countries and varies in the context of Chinese culture for the following reasons. First, some items may not be fully applicable to Chinese healthcare organizations. Such as item 5 [“are there communication standards at your hospital which ensure that patients truly understand the necessary information (e.g., translators, allowing pauses for reflection, calling for further queries)?”] and item 4 [“is individualized health information used at your hospital (e.g., different languages)?”]. Both items refer to providing translation services or health materials in patients’ native languages. However, there are very few immigrants in China, so HLHO-10-C may not be applicable in the country. Second, in recent years, China has focused on constructing “Internet hospitals.” The Internet hospital means applying Internet technology to the provision of health services and information by healthcare organizations, which is similar to telemedicine. It allows healthcare professionals to remotely provide medical services for patients, such as online consultation, prescription, and drug dispensation (39). HLHO-10-C may not evaluate the complexity of “Internet + Medical services” and the accessibility of Internet-based medical navigation devices (e.g., hospital navigation apps). Evaluating the Chinese policies and the current evaluation and practice of HLE in healthcare organizations, it is clear that there is an urgent need to develop a suitable tool for HLE, by evaluating and modifying the existing Health Literacy Environment Scale (HLES) to suit the Chinese healthcare organizations. The person-centered care (PCC) framework proposed by Greene et al. (40) reflects the personal, clinical, and structural dimensions of the patient experience. Therefore, we hypothesize that the PCC framework can be used to construct the HLES to accurately evaluate HLE from the perspective of patients. The purpose of this study was to (1) develop the HLES in China to evaluate the extent to which healthcare organizations and medical professionals can make health information and health services easily obtainable, processable, and understandable for patients, and (2) test its construct validity, content validity, internal consistency reliability, and test–retest reliability.

## 2. Materials and methods

### 2.1. Phase I: definition of domains and designing of initial items

**Figure 1:**
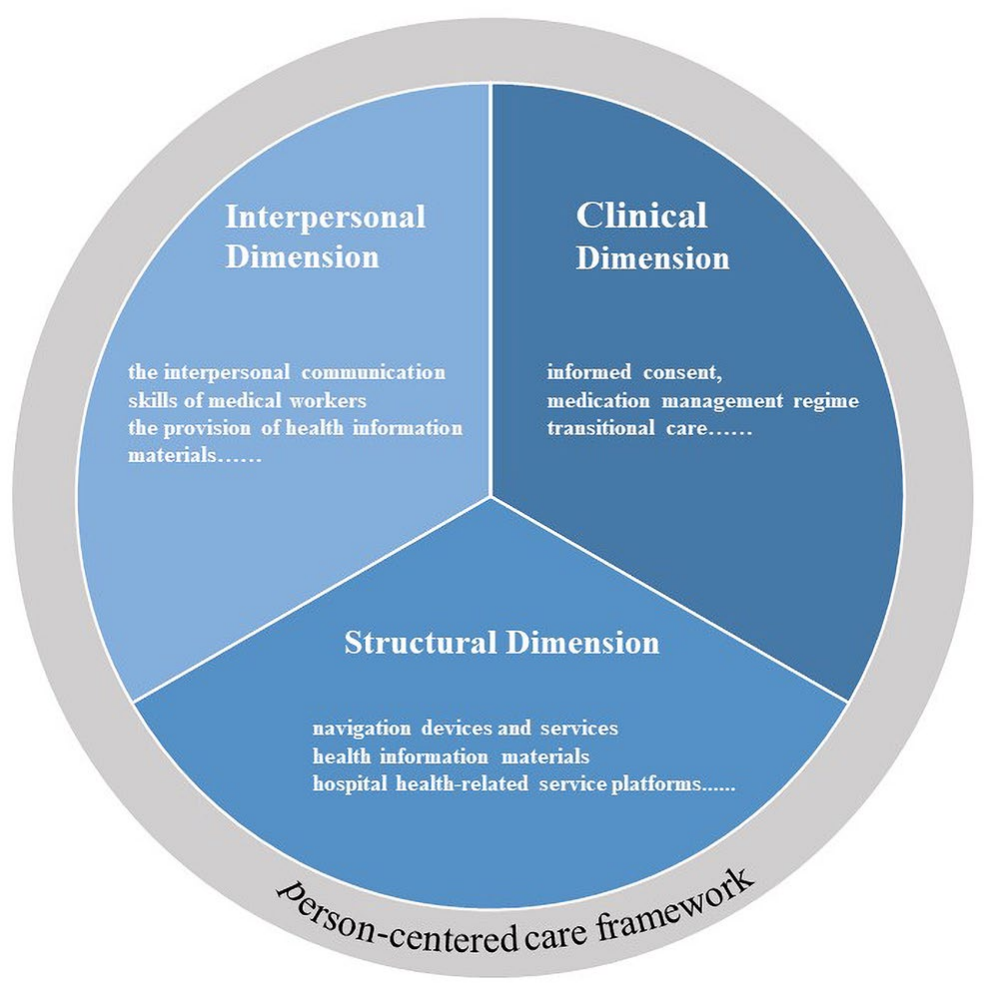
Person-centered care framework in this study.

We used the PCC framework (40) as a theoretical framework for this study. The PCC framework attaches importance to the interaction between patients and the medical environment, in addition to focusing on patient-clinician interactions. The PCC framework has demonstrated good applicability in several studies and practices. To make the PCC framework practically applicable to various healthcare settings (such as healthcare organizations, private clinics, and emergency care centers), Greene et al. (40) classified it into three–interpersonal, clinical, and structural dimensions. The interpersonal dimension refers to the relationship between the service provider and the patient as well as their family members, and the interpersonal communication between them. The clinical dimension focuses on the way health services are delivered, particularly the process of making the clinical decision and providing coordinated and continuous services. The structural dimension involves existing facilities and equipment in the built environment where health services are provided, enhancing the accessibility of health information technology that patients use, and improving procedures to facilitate access to care. The PCC framework is closely connected to HLE construction, that is, focusing on the interaction between patients and the medical environment in the process of obtaining, receiving, and using medical services. At the same time, the PCC framework has been applied in several studies. For example, Prevost (41) used the PCC framework as theoretical guidance to design research tools and a cross-sectional observational investigation of patient perspectives on the accessibility of community paramedicine. Yuliati et al. (42) used the PCC framework to explore the role of case managers in patient-centered care and problems encountered. Therefore, this study used three dimensions–“structural,” “interpersonal” and “clinical” –as the basic structure to evaluate HLE in healthcare organizations. Figure 1 shows the PCC framework that inspired this study, and Table 1 contains a list of the domain definitions of the PCC framework used in our study.

**Table 1:** The domains’ definitions of the HLES.

Domains	Definition
Interpersonal dimension	The relationship between the service provider and the patient as well as their family members, and the interpersonal communication between them, such as the interpersonal communication skills of medical workers and the provision of health information materials.
Clinical dimension	In the process of delivering medical service, medical workers provide professional support and continuous care aimed to improve the accessibility and availability of medical information, such as paying attention to the health literacy of the population under high-risk situations (informed consent, medication management regime that may cause serious consequences, transitional care, and other clinical sessions), and ensuring that patients clear what medical insurance covers and what individuals have to pay.
Structural dimension	Navigable existing facilities and equipment in built environments of healthcare organizations, accessible health information platforms relying on information technology, as well as procedures to promote the acquisition of health services and information, such as navigation devices and services, health information materials, and hospital health-related service platforms.

After determining the dimensions of the HLES, we generated items from the following four aspects. (1) Referring to current HLE-related evaluation tools that have adequate reliability and validity or have been widely used, items were extracted and modified in the following seven tools: Health Literacy-Sensitive Communication Scale (HL-COM), Health Literate Primary Care Practice Screener (HLPC), CAHPS® Health Literacy Item Set for Hospitals, Communication Climate Assessment Toolkit (C-CAT), The Health Literate Health Care Organization 10 Item Questionnaire (HLHO-10), Health Literacy Environment of Hospitals and Health Centers (HLEHHC) and Organizational Health Literacy Responsiveness self-assessment tool (Org-HLR Tool). (2) We searched for the following keywords referring to “health literacy environment,” “organizational/organizational health literacy,” “health literate (health care) “organization/organization.” The search was performed using PubMed, Web of Science, Embase, EBSCO, and Chinese databases such as National Knowledge Infrastructure (CNKI), Wanfang database, and China Science and Technology Journal database. We identified a total of 53 publications related to HLE (2 articles in Chinese and 51 articles in English). We extracted the content related to the construction of HLE to compile items. (3) We conducted qualitative interviews with hospital staff as well as patients and their families to explore the problems and improvement strategies of patients to obtain, understand, and use health information and medical services, to compile new items. Medical workers, patients, and patients’ family members were recruited by purposive sampling. Medical workers with over 3 years of work experience and who engaged in hospital management, health education, and health promotion, or clinical/nursing work were interviewed. The inclusion criteria of patients were: inpatients in internal medicine or surgery, aware of their condition, and able to communicate and comprehend. The inclusion criteria of the patients’ family members were: aware of their condition, over 18 years old, able to communicate and comprehend; the exclusion criteria were: patients who were due to be admitted in the day of the interview; not participating in the accompanying process. A total of 23 participants (11 hospital staff, 9 patients and 3 family members) were included in the study. (4) Items were proposed based on the researcher’s clinical experience.

An expert panel was established to develop an item pool and screen items preliminary. The experts had a minimum of 5 years of working experience, majoring in the fields of health management, hospital administration, and clinical medicine and nursing, with intermediate titles or senior titles. We organized two rounds of focus groups with seven experts. In the first round, the experts wrote and reviewed items and construct the scale pool. Then, each item was classified into three dimensions. In the second round, the experts combined similar items and removed items that are unrelated to HLE or could not be evaluated from the patient’s perspective. Finally, we developed the initial scale containing a total of 57 items, including 16 items on structural dimensions, 16 items on clinical dimensions, and 25 items on interpersonal dimensions. The detailed item content and item source are shown in Appendix 1.

### 2.2. Phase II: development and evaluation of the HLES

This part included four steps: (1) revising the initial scale through Delphi to form the HLES-1 (the draft of pre-test); (2) revising the HLES-1 through pre-test to form the HLES-2 (the draft of pilot test); (3) screening items through pilot test and developing the HLES (final version); and (4) Testing the reliability and validity of the HLES.

#### 2.2.1. Formed HLES-1 (the draft of pre-test) by Delphi

Using the Delphi method, we selected experts to evaluate and revise the initial scale to form the HLES-1 (the draft of pre-test). To meet the inclusion criteria, the experts had to have: (1) A minimum of 5 years of working experience in hospital administration, health education and health promotion, health management, clinical medicine, or nursing; (2) Intermediate or above professional titles. To obtain an adequate number, we invited at least 15 experts (43).

We compiled the initial scale with the expert consultation form and invited experts to fill it out. The contents of the expert consultation form included: (1) the five-point Likert Scale to rate the importance of each item with “1” indicating “not at all important” and “5” indicating “very important.” The experts were invited to modify, delete, or add items in the scale; (2) the experts’ demographic characteristics, and (3) the experts’ judging basis and familiarity with the theme of our study. Depending on the consultation, we conducted 2–3 rounds of meetings with the experts. The Item Importance Score Mean (M) ≤ 4.00 or Coefficient of Variation (CV) ≥ 0.25 was deleted. Finally, we eliminated, adjusted, integrated, and added items to form the HLES-1 (the draft of pre-test) based on the expert opinions and suggestions.

#### 2.2.2. Developed HLES-2 (the draft of pilot test) by pre-test

The pre-test was conducted in July 2021 in three tertiary hospitals–the highest among a three-tier grade system for hospitals in Hangzhou, Zhejiang Province. The tertiary hospital is a medical services center within the region and plays a key role in the medical service system (44). It combines medical treatment, medical education, scientific research, and health prevention. In this study, a 2,400-bed tertiary general hospital, a 1,600-bed tertiary general hospital, and a 600-bed tertiary cancer hospital were included.

Considering that inpatients have more experience with medical services than outpatients and can evaluate HLE more accurately and comprehensively, we decided to select inpatients as study participants. We adopted a convenience sampling method in recruiting at least 20 patients for the pre-test. The inclusion criteria were: patients who were due to be discharged on the day of the survey or who had been in hospital for over 3 days; patients proficient in communication and comprehension. In this session, we used the HLES-1 as the research tool. Each item of the HLES-1 includes five responses consisting of a four-point Likert Scale level scale that includes strongly agree (4), agree (3), disagree (2), and strongly disagree (1), and another response (“not applicable”) without scoring. We used field surveys to investigate patients one to one. After investigation, we asked the patients if there were any unclear expressions, ambiguity, and incomprehensibility in the questionnaire. Referring to the patients’ feedback, we adjusted the scale items to form the HLES-2 (the draft of pilot test).

#### 2.2.3. Developed HLES (final version) by a pilot test

The pilot test was conducted between August 2021 and January 2022. We adopted the same convenience sampling method and inclusion criteria used in the pre-test to recruit patients from the same three tertiary hospitals. Considering the requirements for factor analysis (45), the sample size was 10 times the number of items. In addition, some patients were unable to provide complete answers; therefore, we added about 20% of anticipated invalid samples.

Patients filled in the following three questionnaires: (1) Demographic information questionnaire, which included information on gender, age, marital status, education, monthly household income, residence, competent dialect, the number of hospital admissions, unit admitted to, and length of hospital stay. (2) HLES-2: The responses are the same as for the HLES-1. The total score is calculated by referring to the European health literacy survey (HLS-EU) carried out by the WHO regional office for Europe, and the original score of the scale is converted into the standard score. Appendix 2 shows the calculation method of the scale. (3) The Chinese version of Brief Health Literacy Screen (BHLS-C): BHLS-C was translated by Xue et al. (46) in 2022. The scale combines three of the optimal questions. Three questions are scored from 1 to 5. The total score of the BHLS-C adds each question’s score together, ranging from 3 to 15. Patients with scores below 10 are identified as having inadequate health literacy, and patients with scores from 10 to 12 are identified as having marginal health literacy. The internal consistency reliability of BHLS-C was 0.742 and the criterion validity was 0.519.

We used field surveys to investigate patients in the hospital ward. Based on the results, we used item screening and factor analysis methods to develop the HLES (final version).

#### 2.2.4. Test of the reliability and validity of the HLES

First, to analyze content validity, 16 experts majoring in health management, hospital administration, health education and health promotion, clinical medicine, and nursing were invited. The experts were asked to rate the relevance of each item using a four-point Likert scale (1 = not relevant, 4 = very relevant). Second, the internal consistency reliability and construct validity of the HLES were tested based on the results of the pre-test. In addition, to evaluate the test–retest reliability, 30 patients were randomly selected, and the questionnaire was administered on them twice within a two-week interval.

### 2.3. Statistical methods

Descriptive statistics were used to describe the sample characteristics and calculate the preference percentages. The homoscedasticity of variances among groups was contrasted with Levene’s test. Student t-tests or Mann–Whitney *U* tests were used to compare mean values between two groups. One-way analysis of variance or Kruskal–Wallis tests were applied for the comparison of three or more independent mean values, based on whether there was a normal distribution or not.

First, frequency distribution, item discrimination test, inter-item and item-total correlations, and exploratory factor analysis (EFA) were conducted to reduce the number of items and identify the final version of the HLES.

The criteria for item screening were as follows: (1) Items with missing scores > 20% were considered for elimination (47); (2) using an upper 27%-lower 27% method, the items with no statistically significant difference between upper and lower were deleted (47); (3) items with Pearson r ≤ 0.4 between the score of items and the total score of the HLES were deleted (48); (4) using principal component analysis and varimax-rotation method, common factors were generated and items with factor loading <0.4 were considered for elimination (49).

Second, we tested the validity of the HLES. Construct validity was evaluated by using confirmatory factor analysis (CFA). The criteria for model fit used were: Kline (50) recommended that *χ*^2^/df in ranges of 1 to 3, Tucker Lewis index (TLI), comparative fit index (CFI), goodness-of-fit index (GFI), and adjusted goodness-of-fit index >0.9 as indicators of a good fit. Parsimony goodness of fit index (PGFI), parsimonious normed fit index (PNFI), and parsimony comparative fit index (PCFI) > 0.5 (51). Root mean square error of approximation (RMSEA) and root mean square residual (RMR) should be less than 0.08 for a good model fit (52). Content validity includes item-content validity index (I-CVI) and the scale-content validity index (S-CVI). I-CVI should be >0.78 and S-CVI > 0.90 (53, 54). Finally, we tested the reliability of the HLES. The internal consistency reliability was evaluated using Cronbach’s α (55), and the threshold was 0.70 or greater. Test–retest reliability was conducted on 30 patients who completed the questionnaire twice within a two-week interval. The intra-class correlation coefficient (ICC) was also calculated and classified as follows: 0.5–0.74 as moderate, and 0.75–1 as almost perfect (56).

All data were analyzed at a 95% significance level using IBM SPSS 26.0 and AMOS 24.0.

### 2.4. Ethical approval

Approval to conduct this study was obtained from the Bioethics Committee of Hangzhou Normal University (Grant ID: 2022029).

## 3. Results

### 3.1. Experts’ demographics and results of their consultation

A total of 16 experts participated in the consultation. A total of 10 experts (62.5%) had master’s degrees or above, 11 experts (68.8%) had senior professional titles, and 12 experts (75%) had proficient experience (at least 10 years) in clinical medicine, nursing, hospital administration, or health management. The response rates of the two rounds were 100 and 84.6%, respectively. The expert judging basis coefficient (Ca) and familiarity coefficient (Cs) were 0.8600 and 0.9125, respectively. Therefore, the expert authority coefficient (Cr) was 0.886. The consistency judgment coefficient (Kendall’s W) of the experts for the first and second rounds were 0.186 and 0.234, respectively (*p* < 0.001).

The first round of expert consultation (15 experts) evaluated the importance of the initial scale (57 items). The table of item importance score mean is shown in Appendix 1. There are 13 items with item importance score mean (M) ≤ 4 or CV ≥ 0.25. Meanwhile, the items were added, modified, or deleted based on the experts’ suggestions. The first round of consultation results was as follows: 15 items were deleted, 24 items were modified, and 8 items were added. In the second round of expert consultation (13 experts), the item importance score mean (M) ranged from 4.182 to 5.000, and CV ranged from 0 to 0.171. All items satisfied the item importance score mean (M) > 4 or CV < 0.25. The second round of consultation results was as follows: two items were deleted and eight items were modified. Finally, the HLES-1(48 items) was developed for the pre-test.

### 3.2. Patients’ demographics and results of the pre-test

A total of 20 patients with a mean age of 40.4 years (SD = 13.9) were included; 11 patients were men and 9 patients were women; 11 patients had high school degrees; 10 patients were selected from the internal medicine department and another 10 patients were selected from the surgery department. According to the patients’ feedback, four items were modified and one item was deleted. Finally, the HLES-2 (47 items) was developed for the pilot test.

### 3.3. Patients’ demographics and results of item screen in the pilot test

In total, 697 patients completed the survey, yielding a 96.0% (697/726) response rate. Among them, the majority (60.0%; 418/697) of the patients were investigated in the internal medicine department; 55.1% (384/697) were first hospitalized patients; the median length of hospital stay was 5 days, and 342 (49.1%) patients were hospitalized for 3–7 days. The mean age of patients was 53.5, ranging from 16 to 91 years; 384 (55.1%) were men and 92% were married; the majority of patients (39.0%) had an educational level with at least a junior high school degree. The monthly household income (CNY) of 279 (40.0%) patients ranged between 5,001 and 10,000 yuan. A total of 362 (51.9%) patients were living in the city; 283 (40.60%) patients were accustomed to only speaking their local dialect in daily lives. The results of the BHLS showed that 31.5% of the patients had inadequate health literacy, 23.7% of the patients had marginal health literacy, and 44.8% of the patients had sufficient health literacy.

A total of 10 items that did not meet the criteria were deleted through frequency distribution, item discrimination test, and inter-item and item-total correlations. The detailed results of the item screening above are shown in Appendix 3. The sample of 697 was randomly split into two samples, one for EFA (*n* = 325) and one for CFA (*n* = 372). EFA was performed on the remaining 37 items and the results showed that Kaiser-Meyer-Olkin = 0.955, and *χ*^2^ = 9014.008 of Bartlett’s test (*p* < 0.001), indicating that EFA was suitable.

The results of the screen test and parallel analysis showed that extracted three common factors were the most suitable, and seven items that had a factor loading lower than 0.40 were further eliminated. The factor analysis of the remaining 30 items produced three latent variables, accounting for 59.8% of the cumulative variance contribution rate. The characteristic root values were 6.490 (“structural dimension”), 6.004 (“clinical dimension”), and 5.461(“interpersonal dimension”), and the factor loading was between 0.403 and 0.816. The results of EFA are shown in Table 2. The final version of the HLES had 30 items remaining.

**Table 2:** Results of the third EFA (*n* = 325).

Title item		Principal components		
		1	2	3
S1	Signs or route maps on the outside of the hospital helped me navigate or reach the hospital smoothly	0.638		
S2	Floor indexes, signposts, arrows, text, and other directions to different departments (e.g., Emergency departments, pharmacy, CT, etc.) helped me navigate smoothly	0.599		
S4	When I was hospitalized, I was taken by staff for examination and returned to my ward without navigating by myself	0.413		
S6	I could make an appointment online, by phone, or on-site	0.592		
S7	The hospital arranged the examination in a reasonable order, so I did not have to wait a long time	0.625		
S8	Medical workers informed or directed me to consult and seek guidance remotely via mobile phone	0.781		
S9	Navigator helped me solve the problems (such as informing the place of examination and selecting the department for medical treatment.)	0.633		
S11	Hospital information platforms (e.g., the hospital’s WeChat official accounts platform and app) were designed to make it easy to find or use the functions I need, such as finding out what disease doctors specialize in treating, checking examination reports, and browsing health information	0.664		
S14	Health materials were available in a variety of formats (e.g., prints, drawings, videos, models) that allowed me to absorb health information in a preferred way	0.805		
S15	Health materials (e.g., brochures, posters, videos, and QR codes.) promoted health	0.778		
S16	The content of health materials (e.g., brochures, posters, videos, and QR codes) was easy to understand	0.793		
C1	Medical workers provided me with the disease information adequately		0.758	
C2	Medical workers explained the important results of the examination to me		0.606	
C3	Medical workers discussed with me the information about available treatment options (e.g., desired effect, risks, and costs) in order to involve me in decision-making		0.730	
C4	When signing medical documents (e.g., informed consent form, admission instructions), medical workers made everything clear		0.748	
C6	Guidance from medical workers (e.g., verbal communication, provision of medication labels) helped me take my medication correctly		0.522	
C7	Medical workers let me know emergency symptoms		0.585	
C8	Guidance from medical workers helped me obtain knowledge and skills to keep healthy (e.g., methods of self-monitoring my condition, proper diet and exercise, rehabilitation exercises, etc.)		0.569	
C9	Before the treatment, medical workers told me the approximate cost of the medical treatment		0.725	
C13	Medical workers gave me contact information so that I could contact them for medical help when needed		0.586	
I6	Medical workers had sufficient time for me to consult			0.816
I9	Medical workers encouraged me to ask questions			0.789
I10	Medical workers used simple language to explain medical information to me (e.g., using layman’s terms and explaining medical terms through analogies)			0.403
I11	Medical workers asked me to explain back to them what they had told me (e.g., notice for examination/procedures, health instructions) to make sure I understood it			0.656
I12	When communicating about my condition, treatment, and health education, the medical workers gave me relevant written information (e.g., lists, brochures, and messages)			0.685
I14	When I finished communication, medical workers asked me if I had any other questions			0.442
I15	Medical workers asked me what I had learned about the disease before they gave me health advice			0.746
I16	Medical workers provided me with health information proactively (e.g., brochures, cards, and videos)			0.787
I17	Medical workers highlighted key information in the health materials to me (e.g., through verbal emphasis and marking)			0.429
I20	Medical workers used tools such as images or physical models to explain my condition or provide health advice			0.579

### 3.4. Results of reliability and validity of the HLES

**Figure 2:**
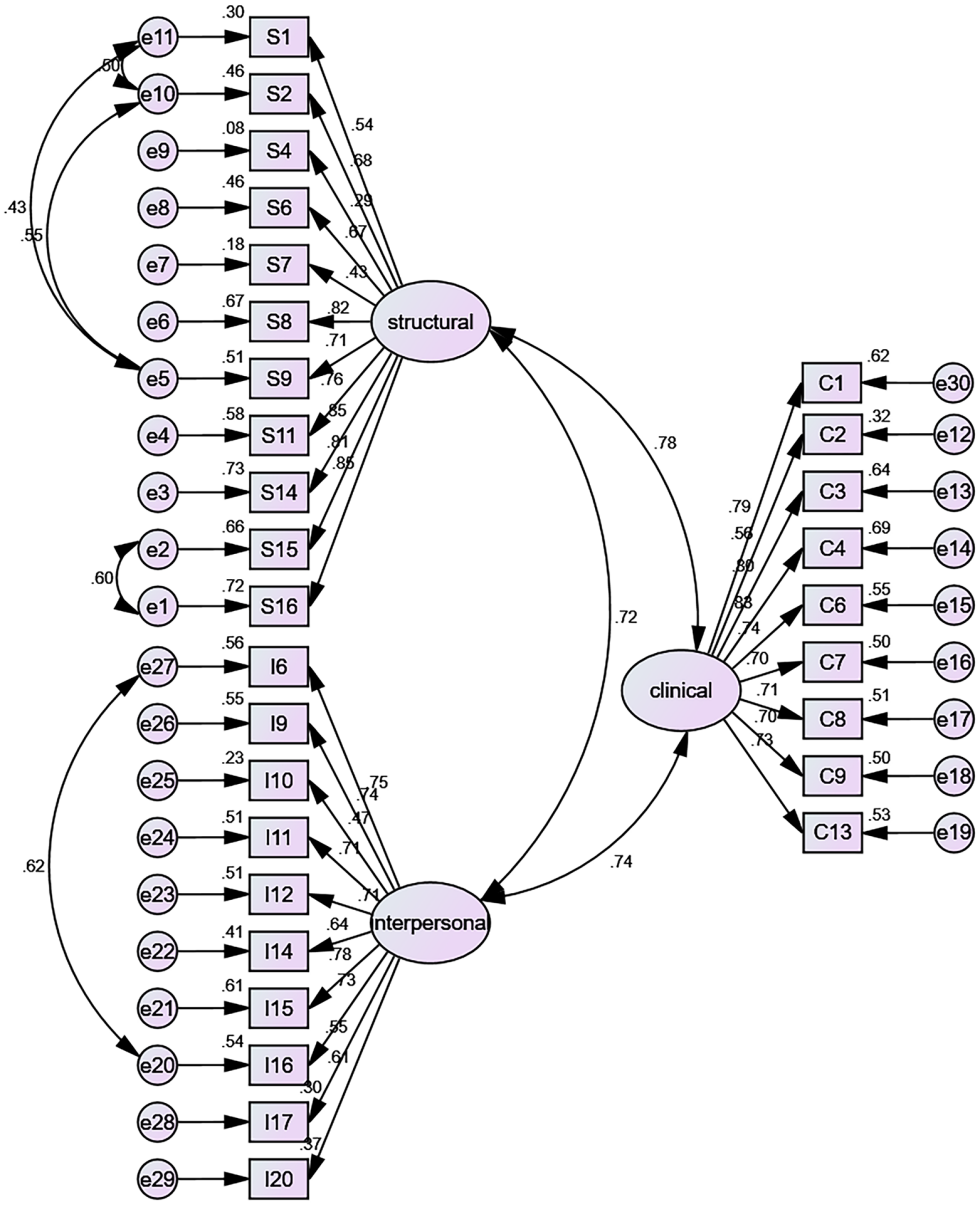
Confirmatory factor analysis.

The validity includes construct validity and content validity. CFA tested each of the three dimensions after conducting EFA to test construct validity. All 30 items were used as observed variables, and the CFA of the three-factor model (Figure 2) showed an acceptable fit after allowing for the correlation of five pairs of error terms (S15-S16, S2-S9, S1-S9, S1-S2, I6-I16). The full model exhibited enough fit statistics (*χ*^2^/df = 2.766, RMR = 0.053, RMSEA = 0.069, CFI = 0.902, IFI = 0.903, TLI = 0.893, GFI = 0.826, PNFI = 0.781, PCFI = 0.823, PGFI = 0.705). The I-CVI of each item in the HLES ranged from 0.91 to 1.00, and the S-CVI/Ave of the total scale was 0.90.

The reliability of the scale was evaluated using two procedures, internal consistency and test–retest (Table 3). The Cronbach’s α for the HLES was 0.960, and the Cronbach’s α for the dimensions were 0.892 (“interpersonal dimension”), 0.915 (“clinical dimension”), and 0.923(“structural dimension”). The ICC between the test and retest ranged from 0.661 to 0.721 in three dimensions, with ICC = 0.844 for the overall scale.

**Table 3:** The results of internal consistency and test–retest.

Dimensions	Item	Cronbach ‘s α	Test–retest
Interpersonal dimension	10	0.892	0.702**
Clinical dimension	9	0.915	0.661*
Structural dimension	11	0.923	0.721**
total scale	30	0.960	0.844**

*p < 0.05; **p < 0.01.

### 3.5. The score of the HLES in the three hospitals

The average score of the HLES was 59.3 ± 20.1 in total for the three hospitals. The three dimensions’ average scores were 44.2 ± 22.2, 67.2 ± 21.6, and 66.8 ± 23.1 for interpersonal, clinical, and structural dimensions, respectively. Hospital A (63.51 ± 18.60) had the highest score in the HLES out of the three hospitals, while hospital C (56.51 ± 21.32) had the lowest score. The total score and dimension score of each hospital are shown in Table 4. As shown in Table 5, we found that the differences in the HLES scores according to patients’ age, education, marital status, monthly household income, residence, competent dialect, and health literacy were statistically significant (*p* < 0.05).

**Table 4:** The scores of the HLES in three hospitals.

Hospital	N	HLES		*F*-value	value of *p*
		Mean	SD		
A	251	63.51	18.60	8.871	<0.001
B	257	57.27	19.87		
C	189	56.51	21.32		

**Table 5:** Demographic characteristics of patients and the difference with the HLES.

Category		Patients (*N* = 697)	The score of HLES (M ± SD)	*F*-value/*t*-value	*p*-value
Ages (years)	≤35	99	72.2 ± 13.0	40.729^a^	<0.001
	36 ~ 45	95	68.9 ± 16.0		
	46 ~ 55	153	61.3 ± 18.1		
	56 ~ 65	195	53.7 ± 20.5		
	>65	155	50.2 ± 20.2		
Gender	Male	384	59.7 ± 19.6	0.529^b^	0.597
	Female	313	58.9 ± 20.7		
Education	Primary school and below	206	42.5 ± 18.4	119.938^a^	<0.001
	Junior middle school	219	60.3 ± 16.4		
	High school	127	68.2 ± 13.6		
	Associated college and above	145	73.9 ± 14.3		
Marriage	Married	641	58.1 ± 19.8	5.632^b^	<0.001
	Unmarried	56	73.5 ± 17.7		
Monthly household Income (CNY)	≤2000 yuan	41	30.5 ± 18.4	172.015^a^	<0.001
	2001 ~ 5,000 yuan	265	47.3 ± 15.9		
	5,001 ~ 10,000 yuan	279	68.1 ± 13.8		
	>10,000 yuan	112	76.3 ± 13.8		
Residence	Rural	335	50.9 ± 19.8	11.608^b^	<0.001
	Town	362	67.1 ± 16.9		
Competent dialect	Mandarin	249	68.0 ± 17.2	71.434^c^	<0.001
	Dialect	283	49.6 ± 19.8		
	Bilingual	165	62.9 ± 17.0		
Department	Internal medicine	418	58.2 ± 20.6	1.849^b^	0.065
	Surgery	279	61.0 ± 19.1		
The number of hospital admissions	First time	384	58.1 ± 20.6	1.509^c^	0.222
	Second time	122	61.7 ± 17.0		
	Third and above	151	58.3 ± 21.6		
Length of hospital stays (days)	<3	86	61.0 ± 20.9	2.506^c^	0.082
	3 ~ 7	342	59.5 ± 19.5		
	>7	169	55.8 ± 21.9		

^a^Welch *F*-value; ^b^*t*-value; ^c^*F*-value.

## 4. Discussion

In this study, we developed the first scale for HLE that specifically reflected Chinese characteristics and had good validity and reliability. The HLES was developed based on the classical research procedure, and it was compiled and modified based on the unique local culture in China.

We tested the HLES for construct validity, content validity, internal consistency reliability, and test–retest reliability. EFA and CFA were used to evaluate the construct validity of the HLES. (1) Three common factors were produced by the EFA, that is, the “structural,” the “clinical,” and the “interpersonal” dimensions. These dimensions’ characteristic root values were approximately 6, and the cumulative variance contribution rate was 59.8%. CFA was tested using structural equation modeling after conducting EFA. Except for the possible small sample size effect, the TLI and GFI was slightly less than the standard (57); other indexes were suitable, indicating that the model had an acceptable fit. The result shows that, overall, the test structure fits well with the default model, and the theoretical construction of the scale is reasonable. In addition, some error terms were correlated. This may be due to the relatively similar measurement content or expression of the items. For example, the error correlation between items S1/S2/S9 may be because the three items are designed to evaluate the navigation of the hospital and the navigation services of medical workers. The error correlation between S15/S16 may reflect the design of health information materials and use the same question stem; (2) We invited experts that majored in health management, hospital administration, health education, health promotion, clinical medicine, and nursing to evaluate the items. The analysis showed that the I-CVI > 0.78 and S-CVI > 0.90, indicating that the content validity of the scale was reliable; (3) The results showed that the Cronbach’s α coefficient for the overall score was 0.960 and ranged between 0.892 and 0.923 for the domains, and (4) the test–retest reliability coefficient for the overall score was 0.844. These findings indicate that the scale had excellent reliability.

The theoretical framework of the scale was PCC, which is classified into three dimensions: interpersonal, clinical, and structural. In addition to current HLE-related measurement tools and content related to the construction of HLE from the literature, we also developed the items based on qualitative research and our own experience. For example, (1) the development of “Internet + Medical” in China. As of June 2021 (58), the internet penetration rate in China reached 74.4%, and the number of internet users in the country reached 1.05 billion, with about 99.6% of the citizens surfing the internet through mobile phones. This shows that the internet and mobile phone usage has become a carrier for Chinese patients in medical treatment and in promoting health information. Patients can make an appointment for registration, payment, and inquiry using their mobile phones (59, 60). Moreover, they can communicate with healthcare professionals for medical consultations, and even browse disease awareness videos online on their mobile phones. Therefore, we added three items (S6, S8, and S11), accounting for 1/10 of the total items, to evaluate the complexity of navigation and appointment services, as well as the accessibility of obtaining health information. Meanwhile, there are three items (S14, S15, and S16) that specifically highlight the comprehensibility and usefulness of health videos and other material obtained by scanning QR codes. (2) Different from other countries, medical resources are relatively constrained in China owing to the country’s large population. From 2018 to 2021, Chinese hospital beds (per 1,000 people) were 6.0, 6.3, 6.4, and 6.7 (61–63). While the data from the World Bank showed (64) that there were 8.0 hospital beds (per 1,000 people) in Grammy in 2017 and 13.0 hospital beds (per 1,000 people) in Japan in 2018. To solve this problem, Chinese hospitals have been built on an increasingly large scale in recent years. According to China’s national health programs development statistical communiqués in 2021 (62), there are 36,570 hospitals in China, with over 4,232 (11.6%) 500-bed hospitals as of 2021. Although to a considerable extent, the expansion of hospital scale can solve medical resource constraints, it also adds complexity for patients to access medical services. The visiting time of outpatients in China is longer than that in other countries. The mean visiting time of outpatients in China was 33 min in 2020 (65), in comparison to 14.5 min in the United States in 2012 (66), 17 min in Japan in 2013 (67), and 15 min in England in 2005 (68). In addition, most of the time spent on outpatient visits in China is spent on navigating the hospital and waiting to be seen, while the time spent communicating with the doctor is very short. Therefore, in the development of HLE, optimizing navigation services is a challenge for Chinese healthcare organizations. In this study, five items (S1/S2/S4/S7/S7), accounting for 1/6 of the total items, were compiled to evaluate the complexity for patients to access medical services in the physical environment, the equipment of navigation staff, and navigation services.

Searching Chinese databases, we found that there is no HLE or related measurement tools. We only found one tool–HLE2–which evaluates the HLE in western countries (14). Patient-reported measures are important in quality improvement efforts because they provide patients’ perceptions of regarding areas of high-quality care and aspects of care that need improvement (69, 70). However, the HLE2 cannot evaluate medical treatments from a patients’ subjective perception, such as the process of doctor-patient communication, informed consent, clinical decision-making, and accessing or browsing health materials. Therefore, it is difficult for the HLE2 to directly evaluate whether patients can successfully access, understand, and use health information and services. However, the HLES, as a patient perspective tool, can effectively evaluate those elements.

In addition, the evaluation contents of the HLES can be used as a reference for Chinese healthcare organizations to practice the indicators proposed in the *Actions to Build a Healthy China (2019–2030)* (31). “Healthcare professionals should actively provide health conduction during treatment” is an advocacy indicator in this action. Healthcare organizations can refer to the interpersonal, clinical, and structural dimensions of the HLES to appraise this indicator. In addition, “establishing the performance appraisal mechanism for health promotion and education conducted by healthcare organizations and professionals” is a binding indicator of the actions. At the same time, how to achieve and appraise this indicator is also the key work of the *Healthy China Initiative* in 2021 and 2022. The HLES can be used as one of the performance appraisal tools to evaluate health promotion and education by healthcare organizations and professionals or to develop performance appraisal indicators.

This study has several strengths. First, the HLES has provided a patient perspective tool to evaluate HLE. Patient-reported measures are arguably one of the best ways to assess constructs that relate to patient-centeredness and evaluate the service quality in healthcare. However, Bremer et al. (71) summarized 13 HLE-related measurement tools in English, and only three of them evaluated HLE from a single patient perspective. Second, most of the strategies for health literacy promotion in China focused on individual competencies where health education is the most common method (30, 72, 73). However, improving health literacy is not just addressed by individual skills and abilities, it also depends on the complexities of the healthcare system (5, 17). This study provides a new perspective on improving health literacy in China, that is, healthcare organizations make it easier for patients to access, understand, and use health information and service, and provides a tool that has good reliability and validity for evaluating the role played by healthcare organizations in health literacy promotion (71). current research about HLE is mainly from developed countries such as the United States, Germany, and Australia, while our study provides research findings from developing countries. The HLES can be used by hospital managers to evaluate HLE regularly, and the results can provide a basis for the establishment and evaluation of quality improvement for health literacy promotion. Managers can formulate targeted health literacy promotion measures based on the three dimensions of HLES–Interpersonal, Clinical, and Structural—by incorporating health literacy promotion into medical workers training and formulating performance appraisal systems to improve medical workers’ ability to practice health literacy (74, 75); formulating regimes for information support and services related to medication management, informed consent and clinical decision-making, and discharge (5); asking for navigation to be considered when designing or renovating health facilities. In addition, policymakers can use HLES to survey the hospitals under their jurisdiction. On the one hand, they can600-obtain benchmark data to issue policies and reduce the differences in HLE between regions; on the other hand, they can adopt targeted improvement countermeasures and provide corresponding resource allocation through the analysis of HLE influencing factors.

## 5. Limitations and future directions

The limitations of this study could be used to develop future research. First, this study was only conducted in Hangzhou, Zhejiang Province, located in the east of China. Although Hangzhou, with adequate medical resources, is representative of HLE measurement (76), subsequent studies need to validate the availability of HLE in other districts (e.g., western, northern, southern, and central China), considering the cultural differences among various ethnic groups and districts in China. Second, this study was conducted only in tertiary hospitals. The availability of the HLES could be subsequently validated in different tiers (e.g., primary and secondary hospitals) or types (e.g., nursing homes, and hospice care centers) of healthcare organizations. Third, a cross-sectional comparison should be conducted across different tiers and types of healthcare organizations to verify the discriminant validity of the HLES. Moreover, some error terms were correlated in CFA, so future research needs to further adjust the content and expression of the items, and expand the sample to test the fitted model. Finally, this study lacks criterion validity. The evaluation of criterion validity needs to use both the “gold standard” scale and the testing scale to measure the concept, but there is no “gold standard” scale of HLE from the patient’s perspective, and similar western scales lack Chinese translation versions and are not fully applicable to China’s situation. Therefore, the criterion validity of the HELS will be further explored in a follow-up study.

## Data availability statement

The datasets used and/or analyzed during the current study are available from the corresponding author on reasonable request.

## Ethics statement

The studies involving human participants were reviewed and approved by Bioethics Committee of Hangzhou Normal University (Grant ID:2022029). The patients/participants provided their written informed consent to participate in this study.

## Author contributions

YT, YiW, and ZX conceived and designed the analysis. YT, YiW, and ZH critically reviewed the article content and wrote the paper mainly. YeW, SL, ZC, and MW were partially involved in writing the paper. ZX and SC collected the data. ZX performed the analysis. YT supervised the project. YT, YiW, and ZH are co-first authors and contributed equally to this manuscript. All authors read and approved the final manuscript.

## Funding

This study was funded by the Soft Science Research Project of Zhejiang Province (Grant No. 2022C35075).

## Conflict of interest

The authors declare that the research was conducted in the absence of any commercial or financial relationships that could be construed as a potential conflict of interest.

## Publisher’s note

All claims expressed in this article are solely those of the authors and do not necessarily represent those of their affiliated organizations, or those of the publisher, the editors and the reviewers. Any product that may be evaluated in this article, or claim that may be made by its manufacturer, is not guaranteed or endorsed by the publisher.

## References

[1] Kindig DA Panzer AM Nielsen-Bohlman L. Health literacy: A prescription to end confusion. Washington, DC: National Academies Press (2004). 25009856

[2] Kutner M Greenburg E Jin Y Paulsen C. The health literacy of America's adults: Results from the 2003 National Assessment of adult literacy (Nces 2006–483). Washington, DC: National Center for Education Statistics (2006).

[3] Sørensen K Pelikan JM Röthlin F Ganahl K Slonska Z Doyle G. Health literacy in Europe: comparative results of the European health literacy survey (Hls-Eu). Eur J Pub Health. (2015) 25:1053–8. doi: 10.1093/eurpub/ckv043, PMID: 25843827 PMC4668324

[4] Rowlands G Shaw A Jaswal S Smith S Harpham T. Health literacy and the social determinants of health: a qualitative model from adult learners. Health Promot Int. (2017) 32:dav093–8. doi: 10.1093/heapro/dav093, PMID: 28180257

[5] Brach C Keller D Hernandez L Baur C Parker R Dreyer B. Ten attributes of health literate health care organizations. NAM Perspect. (2012) 2:1–26. doi: 10.31478/201206a

[6] Champlin S Mackert M Glowacki EM Donovan EE. Toward a better understanding of patient health literacy: a focus on the skills patients need to find health information. Qual Health Res. (2017) 27:1160–76. doi: 10.1177/1049732316646355, PMID: 27179023

[7] Ownby RL Acevedo A Waldrop-Valverde D Jacobs RJ Caballero J. Abilities, skills and knowledge in measures of health literacy. Patient Educ Couns. (2014) 95:211–7. doi: 10.1016/j.pec.2014.02.002, PMID: 24637163 PMC4040019

[8] Benson T Potts HWW Whatling JM Patterson D. Comparison of howRU and eq-5d measures of health-related quality of life in an outpatient clinic. Inform Prim Care. (2013) 21:12–7. doi: 10.14236/jhi.v21i1.9, PMID: 24629651

[9] Zihao X Yingge T Yixue W Siyi C Xuesong Z Meijuan C. Construction of health literate health care organizations and its enlightenment to China. Chin J Hosp Adm. (2021) 37:550–4. doi: 10.3760/cma.j.cn111325-20201208-02193

[10] Clinical Excellence Commission. New South Wales health literacy framework 2019–2024. Sydney: Clinical Excellence Commission (2019).

[11] Schaeffer D Hurrelmann K Bauer U Kolpatzik K. *National action plan health literacy: promoting health literacy in Germany*. (2018). Available at: https://www.nap-gesundheitskompetenz.de/ (Accessed April 21, 2018).

[12] Nutbeam D Muscat DM. Health promotion glossary 2021. Health Promot Int. (2021) 36:1578–98. doi: 10.1093/heapro/daaa157, PMID: 33822939

[13] Parker RM Hernandez LM. What makes an organization health literate? J Health Commun. (2012) 17:624–7. doi: 10.1080/10810730.2012.685806 22571838

[14] Rudd RE Anderson JE. *The health literacy environment of hospitals and health centers. Partners for Action: Making your healthcare facility literacy-friendly*. National Center for the study of adult learning and literacy (NCSALL) (2006).

[15] Australian Commission on Safety and Quality in Health Care. *Health literacy: Taking action to improve safety and quality*. Sydney: Australian Commission on Safety and Quality in Health Care (2014).

[16] Trezona A Dodson S Osborne RH. Development of the organisational health literacy responsiveness (org-Hlr) framework in collaboration with health and social services professionals. BMC Health Serv Res. (2017) 17:513. doi: 10.1186/s12913-017-2465-z, PMID: 28764699 PMC5539902

[17] Santana S Brach C Harris L Ochiai E Blakey C Bevington F. Updating health literacy for healthy people 2030: defining its importance for a new decade in public health. J Public Health Manage Pract. (2021) 27:S258–64. doi: 10.1097/phh.0000000000001324, PMID: 33729194 PMC8435055

[18] Ministry of Health. Health literacy review: A guide. Wellington: Ministry of Health (2015).

[19] Neil S Murphy K Chapman G. Evaluating health literacy environments in Australian health services. Asia Pac J Health Manage. (2018) 13:i35. doi: 10.24083/apjhm.2016.0035

[20] Rudd RE Oelschlegel S Grabeel KL Tester E Heidel E. *HLE2: the health literacy environment of hospitals and health centers*. (2019). Available at: https://cdn1.sph.harvard.edu/wp-content/uploads/sites/135/2019/05/april-30-FINAL_The-Health-Literacy-Environment2_Locked.pdf (Accessed April 30, 2019).

[21] Smith DL Hedrick W Earhart H Galloway H Arndt A. Evaluating two health care Facilities' ability to meet health literacy needs: a role for occupational therapy. Occup Ther Health Care. (2010) 24:348–59. doi: 10.3109/07380577.2010.507267, PMID: 23898960

[22] Oelschlegel S Grabeel KL Tester E Heidel RE Russomanno J. Librarians promoting changes in the health care delivery system through systematic assessment. Med Ref Serv Q. (2018) 37:142–52. doi: 10.1080/02763869.2018.1439216, PMID: 29558326

[23] Palumbo R Annarumma C Musella M. Exploring the meaningfulness of healthcare organizations: a multiple case study. Int J Public Sect Manag. (2017) 30:503–18. doi: 10.1108/IJPSM-10-2016-0174

[24] Park G Kim DH Shao C Theiss LM Smith B Marques IC. Organizational assessment of health literacy within an Academic Medical Center. Am J Surg. (2023) 225:129–30. doi: 10.1016/j.amjsurg.2022.08.004, PMID: 35981910 PMC10468260

[25] Griese L Berens EM Nowak P Pelikan JM Schaeffer D. Challenges in navigating the health care system: development of an instrument measuring navigation health literacy. Int J Environ Res Public Health. (2020) 17:5731. doi: 10.3390/ijerph17165731, PMID: 32784395 PMC7460304

[26] MacLeod S Musich S Gulyas S Cheng Y Tkatch R Cempellin D. The impact of inadequate health literacy on patient satisfaction, healthcare utilization, and expenditures among older adults. Geriatr Nurs. (2017) 38:334–41. doi: 10.1016/j.gerinurse.2016.12.003, PMID: 28089217

[27] Daily C. *China's health literacy improving, commission says*. (2022). Available at: https://www.chinadaily.com.cn/a/202206/08/WS62a0383ba310fd2b29e6169d.html (Accessed June 8, 2022).

[28] Berkman ND Sheridan SL Donahue KE Halpern DJ Viera A Crotty K. Health literacy interventions and outcomes: an updated systematic review. Evid Rep Technol Assess (Full Rep). (2011) 199:1–941. PMC4781058 23126607

[29] Jayasinghe UW Harris MF Parker SM Litt J van Driel M Mazza D. The impact of health literacy and life style risk factors on health-related quality of life of Australian patients. Health Qual Life Outcomes. (2016) 14:1–13. doi: 10.1186/s12955-016-0471-1, PMID: 27142865 PMC4855442

[30] National Health Commission of the People’s Republic of China. *National plan of health literacy promotion initiatives (2014-2020)*. (2014). Available at: http://www.nhc.gov.cn/xcs/s3582/201405/da9eb5932deb4ac1b0ee67ca64d6999e.shtml (Accessed May 9, 2014).

[31] State Council of the People’s Republic of China. *Actions to build a healthy China (2019-2030)*. (2019). Available at: http://www.gov.cn/xinwen/2019-07/15/content_5409694.htm (Accessed July 5, 2019).

[32] Kun X Wenli D. Functions of large public hospitals in public health literacy promoting. Hosp Adm J Chin Peoples Liber Army. (2016) 23:687–8. doi: 10.16770/J.cnki.1008-9985.2016.07.033

[33] Huimin W. Practice and experience of health literacy promotion action in community health service center. J Community Med. (2011) 9:66–8.

[34] Healthy China Initiative Promotion Committee Office. *Circular on publishing the work plan on promoting the implementation of healthy China initiative in 2020*. (2020). Available at: http://www.nhc.gov.cn/guihuaxxs/s3585u/202009/4757048a304b45e49d8073b33df0647d.shtml (Accessed September 1, 2020).

[35] Healthy China Initiative Promotion Committee Office. *Circular on publishing the work plan on promoting the implementation of healthy China initiative in 2021*. (2021). Available at: http://www.nhc.gov.cn/guihuaxxs/s7788/202104/2241e2f8f42e4769aa1c5acc5f0e0ce2.shtml (Accessed April 13, 2021).

[36] Healthy China Initiative Promotion Committee Office. *Circular on publishing the work plan on promoting the implementation of healthy China initiative in 2022*. (2022). Available at: http://www.nhc.gov.cn/guihuaxxs/s7788/202204/67cb879e0afd44ba916912367de56170.shtml (Accessed April 2, 2022).

[37] Tong Y Xue Z Gu L Xia Y Zhang C Huang L. Evaluation of the reliability and validity of the Chinese version of health literate health care organization 10-item questionnaire. Chin J Hosp Adm. (2021) 37:555–9. doi: 10.3760/cma.j.cn111325-20210303-00175

[38] Kowalski C Lee SY Schmidt A Wesselmann S Wirtz MA Pfaff H. The health literate health care organization 10 item questionnaire (HLHO-10): development and validation. BMC Health Serv Res. (2015) 15:47. doi: 10.1186/s12913-015-0707-5, PMID: 25638047 PMC4332719

[39] Zhi LH Yin P Ren JJ Wei GQ Zhou J Wu J. Running an internet hospital in China: perspective based on a case study. J Med Internet Res. (2021) 23:e18307. doi: 10.2196/18307, PMID: 34342267 PMC8485192

[40] Greene SM Tuzzio L Cherkin D. A framework for making patient-centered care front and center. Perm J. (2012) 16:49–53. doi: 10.7812/TPP/12-025, PMID: 23012599 PMC3442762

[41] Prevost C. Patient experiences in rural northern Ontario: Small hospital utilization and perspectives on community paramedicine. Ontario: Laurentian University of Sudbury (2019).

[42] Yuliati NK Nopiyani NMS Duarsa DP. The roles of case managers and problems encountered in implementing patient centered care in hospitals. Public Health Prev Med Arch. (2019) 7:140–7. doi: 10.53638/phpma.2019.v7.i2.p11

[43] Clayton MJ. Delphi: a technique to harness expert opinion for critical decision-making tasks in education. Educ Psychol. (1997) 17:373–86. doi: 10.1080/0144341970170401

[44] Zhang W Deng Z Evans R Xiang F Ye Q Zeng R. Social media landscape of the tertiary referral hospitals in China: observational descriptive study. J Med Internet Res. (2018) 20:e9607. doi: 10.2196/jmir.9607, PMID: 30093370 PMC6107732

[45] Neyman J. Proceedings of the third Berkeley symposium on mathematical statistics and probability: Contributions to astronomy and physics. Berkeley, California: University of California Press (1956).

[46] Xue Z Tong Y Wu Y Chen S Zhang Y Zhu Z. Chinesization of brief health literacy screen and its reliability and validity. J Pract Med. (2022) 29:378–82. doi: 10.3969/j.issn.1006-3110.2022.03.033

[47] Baloglu N Karadag E Karaman H. The strategic planning attitude scale: a study of exploratory and confirmatory factor analyses. Educ Sci. (2008) 8:429–37.

[48] Elsman E van Nispen R van Rens GH. Psychometric evaluation of the participation and activity inventory for children and youth (pai-cy) 0–2 years with visual impairment. Qual Life Res. (2020) 29:775–81. doi: 10.1007/s11136-019-02343-1, PMID: 31673921 PMC7028793

[49] Ware JE Jr Gandek B. Methods for testing data quality, scaling assumptions, and reliability: the IQOLA project approach. J Clin Epidemiol. (1998) 51:945–52. doi: 10.1016/S0895-4356(98)00085-7 9817111

[50] Kline RB. Principles and practice of structural equation modeling. New York: Guilford Publications (2015).

[51] Schreiber JB Nora A Stage FK Barlow EA King J. Reporting structural equation modeling and confirmatory factor analysis results: a review. J Educ Res. (2006) 99:323–38. doi: 10.3200/JOER.99.6.323-338

[52] Hu L Bentler PM. Cutoff criteria for fit indexes in covariance structure analysis: conventional criteria versus new alternatives. Struct Equ Mod J Multidiscip Res. (1999) 6:1–55. doi: 10.1080/10705519909540118

[53] Jais SD. Successful use of information in multinational companies. Berlin: Springer (2007).

[54] McDonald RP Ho M-HR. Principles and practice in reporting structural equation analyses. Psychol Methods. (2002) 7:64–82. doi: 10.1037/1082-989X.7.1.64, PMID: 11928891

[55] Polit DF Beck CT. The content validity index: are you sure you know what's being reported? Critique and recommendations. Res Nurs Health. (2006) 29:489–97. doi: 10.1002/nur.20147, PMID: 16977646

[56] Nunnally JC Bernstein IH. Psychometric theory. 3rd ed. New York: McGraw-Hill (1994).

[57] Shi D Lee T Maydeu-Olivares A. Understanding the model size effect on Sem fit indices. Educ Psychol Meas. (2019) 79:310–34. doi: 10.1177/0013164418783530, PMID: 30911195 PMC6425088

[58] China Internet Network Information Center. *The 50th statistical report on the development status of the internet in China* (2022). Available at: http://www3.cnnic.cn/n4/2022/0914/c88-10226.html. (Accessed August 31, 2022).

[59] Lai Y Chen S Li M Ung COL Hu H. Policy interventions, development trends, and service innovations of internet hospitals in China: documentary analysis and qualitative interview study. J Med Internet Res. (2021) 23:e22330. doi: 10.2196/22330, PMID: 34283025 PMC8335616

[60] Tu J Wang C Wu S. The internet hospital: an emerging innovation in China. Lancet Glob Health. (2015) 3:e445–6. doi: 10.1016/S2214-109X(15)00042-X, PMID: 26187488 PMC7129805

[61] National Health Commission of the People's Republic of China. *China's national health programs developed statistical communiqués in 2020* (2021). Available at: http://www.nhc.gov.cn/guihuaxxs/s10743/202107/af8a9c98453c4d9593e07895ae0493c8.shtml (Accessed July 13, 2021).

[62] National Health Commission of the People's Republic of China. *China's national health programs developed statistical communiqués in 2021*. (2022). Available at: http://www.nhc.gov.cn/guihuaxxs/s3586s/202207/51b55216c2154332a660157abf28b09d.shtml (Accessed July 12, 2022).

[63] National Health Commission of the People's Republic of China. *China's national health programs developed statistical communiqués in 2019* (2020). Available at: http://www.nhc.gov.cn/guihuaxxs/s10748/202006/ebfe31f24cc145b198dd730603ec4442.shtml (Accessed June 6, 2020).

[64] World Bank. *Hospital beds (per 1,000 people)* (2020). Available at: https://data.worldbank.org/indicator/SH.MED.BEDS.ZS (Accessed January 1, 2020).

[65] Wang M He Z Wu Q Zeng X Mai X Peng D. Effect analysis of shortening out-patient waiting time in large public hospitals. Mod Hosp Manage. (2021) 19:24–6. doi: 10.3969/j.issn.1672-4232.2021.06.007

[66] Migongo AW Charnigo R Love MM Kryscio R Fleming ST Pearce KA. Factors relating to patient visit time with a physician. Med Decis Mak. (2012) 32:93–104. doi: 10.1177/0272989X10394462 21393556

[67] Aomatsu M Abe H Abe K Yasui H Suzuki T Sato J. Validity and reliability of the Japanese version of the care measure in a general medicine outpatient setting. Fam Pract. (2014) 31:118–26. doi: 10.1093/fampra/cmt053, PMID: 24115011

[68] Irving G Holden J. 15 minute consultations as standard benefit patients and GPs. BMJ Open. (2012) 344:e3704. doi: 10.1136/bmj.e3704, PMID: 22661722

[69] de Bienassis K Kristensen S Hewlett E Roe D Mainz J Klazinga N. Measuring patient voice matters: setting the scene for patient-reported indicators. Int J Qual Health Care. (2022) 34:ii3–6. doi: 10.1093/intqhc/mzab002, PMID: 33575802

[70] Tzelepis F Sanson-Fisher RW Zucca AC Fradgley EA. Measuring the quality of patient-centered care: why patient-reported measures are critical to reliable assessment. Patient Prefer Adherence. (2015) 9:831–5. doi: 10.2147/ppa.s81975, PMID: 26150703 PMC4484696

[71] Bremer D Klockmann I Jaß L Härter M von dem Knesebeck O Lüdecke D. Which criteria characterize a health literate health care organization? A scoping review on organizational health literacy. BMC Health Serv Res. (2021) 21:664. doi: 10.1186/s12913-021-06604-z, PMID: 34229685 PMC8259028

[72] Xue C Li Y. Hotspots and frontiers of health literacy research in mainland China: bibliometric analysis based on CNKI core journals. NVEO. (2021) 8:7639–48.

[73] Liu X. Effect of health literacy intervention on self-management ability of in-patients with chronic hepatitis B. J Nurs Pract. (2020) 17:54–6. doi: 10.3969/j.issn.1672-9676.2020.22.021

[74] Kaper MS Sixsmith J Koot JAR Meijering LB Van Twillert S. Developing and pilot testing a comprehensive health literacy communication training for health professionals in three European countries. Patient Educ Couns. (2018) 101:152–8. doi: 10.1016/j.pec.2017.07.017, PMID: 28823383

[75] Kaper M Sixsmith J Meijering L Vervoordeldonk J Doyle P Barry MM. Implementation and long-term outcomes of Organisational health literacy interventions in Ireland and the Netherlands: a longitudinal mixed-methods study. Int J Environ Res Public Health. (2019) 16:4812–30. doi: 10.3390/ijerph16234812, PMID: 31795504 PMC6926611

[76] He H Zhu M Liao F Tong F Su L Chen K. Cluster analysis of the distribution of medical resources in Zhejiang. Chin J Hosp Adm. (2006) 3:201–3. doi: 10.3760/j.issn:1000-6672.2006.03.025

