# Supplementary material for: Development and validation of the health literacy environment scale for Chinese hospitals from patients’ perspective
Source: Front Public Health. 2023 Jun 2;11:1130628. doi: 10.3389/fpubh.2023.1130628 (PMC10273272; doi:10.3389/fpubh.2023.1130628)
Supplement: Supplementary file 1 [file Table_1.DOCX]

Supplementary Material

**Development and Validation of the Health Literacy Environment Scale for Chinese Hospitals from Patients’ Perspective**

Yingge Tong^1†*^, Yixue Wu^1†^, Zhiqing Han^1†^, Zihao Xue^2^, Yeling Wei^2^, Shanyuan Lai^1^, Ziyi Chen^1^, Miaoling Wang^1^, Siyi Chen^1^

^1^School of Nursing, Hangzhou Normal University, Hangzhou, China

^2^Department of Operating Room, Affiliated Sir Run Run Shaw Hospital, Zhejiang University School of Medicine, Hangzhou, China

*** Correspondence:**Yingge Tong
[1352597965@qq.com](mailto:1352597965@qq.com)

Yixue Wu
[zhhwyx1130@163.com](mailto:zhhwyx1130@163.com)

Zhiqing Han
[358169822@qq.com](mailto:358169822@qq.com)

Appendix 1. Items of the initial scale and the coefficient of variation for the first round expert meeting

| Dimensions | Items (n= 57) | Sources | M | SD | CV |
| --- | --- | --- | --- | --- | --- |
| Interpersonal | 1 All staff (including doctors, nurses, carers, fee earners, etc.) treat each other and me equally when communicating | clinical experience | 4.733 | 0.442 | 0.093 |
| Interpersonal | 2* Medical staff sometimes make me feel uncomfortable in the way they talk to me or what they say or do | clinical experience | 4.533 | 0.718 | 0.158 |
| Interpersonal | 3* Because of my limited literacy level, I sometimes worry that I will be treated lightly or differently by medical staff | clinical experience | **3.933** | 1.062 | **0.270** |
| Interpersonal | 4 The way the medical staff talked to me made me feel at ease | literature review | 4.067 | 1.062 | **0.261** |
| Interpersonal | 5 The medical staff gave me plenty of time and encouraged me to ask them for advice on things I had questions about | interview | 4.800 | 0.400 | 0.083 |
| Interpersonal | 6* When hospital staff talk to me, they speak too fast for me to understand | interview | 4.733 | 0.772 | 0.163 |
| Interpersonal | 7 Verbal conversation about my condition and treatment, with additional written information provided to me by the medical staff | HL-COM | 4.733 | 0.573 | 0.121 |
| Interpersonal | 8 During the communication process, the medical staff encouraged me to ask questions if I did not understand | HL-COM | 4.800 | 0.400 | 0.083 |
| Interpersonal | 9 At the end of the communication, the medical staff will ask me if there are any other issues to discuss | interview | 4.600 | 0.800 | 0.174 |
| Interpersonal | 10 The medical staff asked me to repeat in my own words what they had told me (e.g., what to prepare for the operation, how to observe my condition, principles of diet) to make sure I could understand it | HLUPT | 4.600 | 0.879 | 0.191 |
| Interpersonal | 11 After conducting health instruction (e.g., deep breathing, elastic stockings, insulin, stoma maintenance), the medical staff asked me to do it all over for them to make sure I really grasped the content | interview | 4.867 | 0.340 | 0.070 |
| Interpersonal | 12 How much knowledge of the disease I currently have is taken into account before the health worker gives me a health education | interview | 4.600 | 0.712 | 0.155 |
| Interpersonal | 13 The medical staff explained my condition in a way that I could understand (e.g. using everyday language, explaining medical terms by analogy) | interview | 4.800 | 0.400 | 0.083 |
| Interpersonal | 14 The medical staff gave me specific, easy to follow health guidance (e.g., "drink 1500ml of water a day" instead of "drink more water", "no more than 6g of salt and 25-30g of cooking oil a day" instead of "less oil and less salt") | interview | 4.600 | 0.611 | 0.133 |
| Interpersonal | 15 The health education materials provided by the medical staff have the knowledge I need most, which will enable me to follow the instructions in my daily life | interview | 4.800 | 0.542 | 0.113 |
| Interpersonal | 16 Health education materials convey just the right amount of information for me | interview | 4.533 | 0.718 | 0.158 |
| Interpersonal | 17 The medical staff will provide me with health education materials (e.g., materials in text form, picture form, video form, physical models) that are suitable for my use, depending on my reading ability or literacy level | Org-HLR | 4.600 | 0.611 | 0.133 |
| Interpersonal | 18 The content of hospital health education materials meets my needs for maintaining and promoting my health | interview | 4.667 | 0.596 | 0.128 |
| Interpersonal | 19 Medical staff have asked me about my experience of using health education materials (e.g. was the content easy to understand, did it help me understand my condition, etc.) | clinical experience | 4.533 | 0.618 | 0.136 |
| Interpersonal | 20 The hospital has various forms of health education materials such as brochures, posters, videos and physical models, so I can choose the way I like to receive health information | interview | 4.333 | 1.135 | **0.262** |
| Interpersonal | 21 The design of the hospital health education materials (e.g., use of illustrations, appropriate font size) makes it easy for me to understand the content of the health education | literature review | 4.333 | 1.135 | **0.262** |
| Interpersonal | 22 Medical staff will inform me of key information in the information by verbally highlighting it, marking it, etc. when I am given written information | literature review | 4.467 | 0.806 | 0.180 |
| Interpersonal | 23 The medical staff will offer to inform or help me watch health education videos on information-based approaches (e.g. help me scan the QR code on the information board on my mobile phone to play the post-operative functional exercise video) | interview | 4.467 | 0.884 | 0.198 |
| Interpersonal | 24 I am satisfied with the process of interacting with the medical staff | interview | 4.067 | 1.289 | **0.317** |
| Interpersonal | 25 I am satisfied with the results of my interaction with the medical staff | interview | 4.200 | 1.222 | **0.291** |
| Clinical | 26 Through talking to the hospital staff, I found out the items and the amount of money I had to pay out of pocket for this treatment | HLHO-10 | 4.200 | 1.108 | **0.264** |
| Clinical | 27 Before treatment began, the medical staff clearly informed me of the medical costs I would have to pay | HLHO-10 | 4.400 | 0.952 | 0.216 |
| Clinical | 28 Before the treatment started, the medical staff told me exactly what was covered by my medical insurance and what I would have to pay for out of pocket | interview | 4.267 | 1.236 | **0.290** |
| Clinical | 29 The medical staff discussed and compared all the treatment options available to me (e.g. expected outcomes, risks and costs) in order for me to make the right choice for my situation | interview | 4.933 | 0.249 | 0.051 |
| Clinical | 30 The doctor was able to discuss the pros and cons of the different treatment options with me, as I would have liked to do | interview | 4.357 | 1.394 | **0.320** |
| Clinical | 31 The medical staff explained the test results fully to me, which helped me to know how my condition was progressing or to make the right decision for my situation | HL-COM | 4.667 | 0.596 | 0.128 |
| Clinical | 32 At the time of signing the consent form, the medical staff had tried to ensure that I understood everything | HL-COM | 4.733 | 0.442 | 0.093 |
| Clinical | 33 When signing, the medical staff explained the contents of the informed consent form to me in detail and fully | interview | 4.467 | 0.884 | 0.198 |
| Clinical | 34 The medical staff explained to me how much of each medication to take, when to take it and the precautions to be taken during and after discharge from hospital. | literature review | 4.667 | 0.869 | 0.186 |
| Clinical | 35 Through the guidance of the medical staff (e.g. verbal advice, drawing a medication schedule, reminding me to use the dispenser box), I was able to take my medication on time after discharge, following the instructions | literature review | 4.467 | 0.806 | 0.180 |
| Clinical | 36 The medical staff will work with me to check how much of each medication I need to take, when to take it and why | literature review | 4.600 | 0.879 | 0.191 |
| Clinical | 37 Through the guidance of the medical staff, I know what symptoms I have when I have an emergency and what I should do | literature review | 4.800 | 0.542 | 0.113 |
| Clinical | 38 Through the guidance of the medical staff, I have acquired knowledge and skills related to the management of my illness (e.g. methods of self-monitoring, proper diet and exercise, rehabilitation exercises, etc.) | interview | 4.800 | 0.542 | 0.113 |
| Clinical | 39 The information on the written discharge instructions for follow-up appointments let me know when and where to go for my next follow-up appointment and the department | interview | 4.933 | 0.249 | 0.051 |
| Clinical | 40 If I need a test after discharge, the doctor will make an appointment for me to have the test I need | interview | **3.467** | 1.258 | **0.363** |
| Clinical | 41 If I need a follow-up appointment after discharge, the doctor will book a follow-up appointment for me and I just need to come to the hospital at the appointed time. | interview | **3.200** | 1.424 | **0.445** |
| Structure | 42 Public transport near the hospital has signs or route maps pointing to the hospital, which helps me get there smoothly | HLEHHC2 | 4.267 | 0.998 | 0.234 |
| Structure | 43 Floor indexes, signposts, arrows, text and other directional information leading to different departments (e.g. Emergency departments, Pharmacy, CT, etc.), which helped me a lot in finding my way around the hospital | literature review | 4.733 | 0.772 | 0.163 |
| Structure | 44 The information on the signs or road signs in the hospital are detailed and clear and help me to find my way around. | interview | 4.667 | 0.699 | 0.150 |
| Structure | 45 The hospital has placed signs where I think directions are needed and they are very clearly placed | interview | 4.800 | 0.400 | 0.083 |
| Structure | 46 When finding my way around the hospital, staff or volunteers will offer to help me (e.g. guide or escort me to where I need to go) | interview | 4.733 | 0.442 | 0.093 |
| Structure | 47 I was taken to the hospital for examination without having to find my way around the hospital | interview | 4.333 | 1.011 | 0.233 |
| Structure | 48* The hospital has a complex layout of buildings and sometimes I couldn't find where I was going | interview | 4.133 | 1.087 | **0.263** |
| Structure | 49* I have to move around the hospital in order to complete activities such as visits to the clinic, laboratory tests, X-ray films and medication collection | interview | 4.067 | 0.998 | 0.2454 |
| Structure | 50 Whether I register online, by phone or on site, I can get the number I need without any problems | interview | 4.467 | 1.024 | 0.229 |
| Structure | 51 Multiple tests I need to have done (e.g. ECG, ultrasound, CT, MRI, etc.) can be done in the same day | interview | **4.000** | 1.265 | **0.316** |
| Structure | 52 The hospital scheduled all the tests for me so I didn't have to spend too much time waiting for each test to be carried out | interview | 4.467 | 1.024 | 0.229 |
| Structure | 53 Medical staff to inform or guide me, I can ask, consult and seek guidance from a doctor remotely via mobile phone | interview | 4.467 | 0.806 | 0.180 |
| Structure | 54 Information devices (e.g. bedside TVs, tablets) for health education content are installed and in service in the ward | literature review | 4.133 | 0.957 | 0.231 |
| Structure | 55 The medical staff will inform or guide me to use the functions on the information platform (e.g. hospital WeChat, official hospital app), such as registering, checking reports, paying bills, requesting copies of medical records, etc. | interview | 4.667 | 0.699 | 0.150 |
| Structure | 56 I think hospital information platforms (e.g. hospital WeChat public website, official hospital app) have a user-friendly interface and are easy to operate, allowing me to search for the information I need easily | interview | 4.800 | 0.400 | 0.083 |
| Structure | 57 When I need to use the self-service machines in the hospital to print out test reports or settle bills, I am given instructions on how to do so | interview | 4.600 | 0.712 | 0.155 |
| Note: M=the mean score of the importance of each item by the 16 experts; SD=Standard Deviation of the importance score of each item; CV= Coefficient of Variation, the calculated formula was used as follows: $CV=\frac{SD}{M}$. | | | | | |

Appendix 2. The calculation method of the scale and examples

In order to normalize the values in a dataset to be between 0 and 100, the Standard Score (SS) calculated formula was used as follows:

$$SS=\frac{X-min}{max-min}\times100$$

*X=the total value of items in the given dataset; *Range(max-min)*=The difference between the highest and lowest values among the given dataset. Examples of the calculations are shown in Table 1. During the factor analysis, the option of “not applicable” was equal to missing data, and replaced missing values of the item with the average of known values for that item.

Table 1. Samples of the standard score (SS) calculated for HLES

| Initial score | | | | | X | max | min | SS |
| --- | --- | --- | --- | --- | --- | --- | --- | --- |
| Item 1 | Item 2 | Item 3 | Item 4 | Item 5 |  |  |  |  |
| 1 | 1 | 1 | N/A | N/A | 3 | 12 | 3 | 0 |
| 4 | 4 | N/A | 4 | 4 | 16 | 16 | 4 | 100 |
| 3 | 3 | 3 | 3 | 3 | 15 | 20 | 5 | 66.7 |
| 1 | 3 | 2 | 3 | N/A | 9 | 16 | 4 | 41.7 |
| Note: a four-point Likert scale was designed to respond; N/A= not applicable | | | | | | | | |

Appendix 3. Result of the item screen

(1) Frequency Distribution

HLES-2 (the draft of pilot test) is composed of 47 items. The answers are given using a Likert scale with five possible choices: strongly agree (4), agree (3), disagree (2), strongly disagree (1), and not applicable (without scoring). As shown in Table 2, the response rate of C12 and C14 on “not applicable” exceeded 40%, demonstrating that patients may have difficulty responding. Thus, these items (I5, S13, C12, and C14) were deleted.

Table 2. Frequency Distribution (*n*=697)

| item | Responses (n, %) | | | | | Missing (n,%) |
| --- | --- | --- | --- | --- | --- | --- |
|  | **Strongly agree** | **disagree** | **agree** | **Strongly agree** | **Not applicable** |  |
| I1 | 1(0.1) | 15(2.2) | 213(30.6) | 468(67.1) | 0(0) | 0(0) |
| I2 | 57(8.2) | 88(12.6) | 177(25.4) | 375(53.8) | 0(0) | 0(0) |
| I3 | 555(79.6) | 127(18.2) | 2(0.3) | 13(1.9) | 0(0) | 0(0) |
| I4 | 552(79.2) | 133(19.1) | 2(0.3) | 10(1.4) | 0(0) | 0(0) |
| I5 | 563(80.8) | 120(17.2) | 5(0.7) | 9(1.3) | 0(0) | 0(0) |
| I6 | 189(27.1) | 341(48.9) | 76(10.9) | 91(13.1) | 0(0) | 0(0) |
| I7 | 35(5.0) | 170(24.4) | 262(37.6) | 230(33) | 0(0) | 0(0) |
| I9 | 219(31.4) | 401(57.5) | 43(6.2) | 34(4.9) | 0(0) | 0(0) |
| I10 | 45(6.5) | 88(12.6) | 226(32.4) | 337(48.4) | 0(0) | 1(0.1) |
| I11 | 151(21.7) | 285(40.9) | 115(16.5) | 146(20.9) | 0(0) | 0(0) |
| I12 | 173(24.8) | 350(50.2) | 92(13.2) | 82(11.8) | 0(0) | 0(0) |
| I13 | 542(77.8) | 130(18.7) | 14(2.0) | 11(1.6) | 0(0) | 0(0) |
| I14 | 116(16.6) | 204(29.3) | 172(24.7) | 204(29.3) | 0(0) | 1(0.1) |
| I15 | 189(27.1) | 394(56.5) | 74(10.6) | 40(5.7) | 0(0) | 0(0) |
| I16 | 176(25.3) | 330(47.3) | 92(13.2) | 99(14.2) | 0(0) | 0(0) |
| I17 | 69(9.9) | 314(45.1) | 172(24.7) | 142(20.4) | 0(0) | 0(0) |
| I18 | 64(9.2) | 161(23.1) | 211(30.3) | 261(37.4) | 0(0) | 0(0) |
| I20 | 166(23.8) | 302(43.3) | 95(13.6) | 134(19.2) | 0(0) | 0(0) |
| C1 | 12(1.7) | 49(7.0) | 314(45.1) | 322(46.2) | 0(0) | 0(0) |
| C2 | 26(3.7) | 70(10.0) | 254(36.4) | 345(49.5) | 2(0.3) | 0(0) |
| C3 | 41(5.9) | 143(20.5) | 240(34.4) | 271(38.9) | 2(0.3) | 0(0) |
| C4 | 47(6.7) | 144(20.7) | 232(33.3) | 267(38.3) | 7(1.0) | 0(0) |
| C5 | 52(7.5) | 158(22.7) | 195(28.0) | 286(41.0) | 6(0.9) | 0(0) |
| C6 | 37(5.3) | 70(10.0) | 272(39.0) | 236(33.9) | 80(11.5) | 2(0.3) |
| C7 | 69(9.9) | 263(37.7) | 190(27.3) | 174(25.0) | 0(0) | 1(0.1) |
| C8 | 55(7.9) | 206(29.6) | 246(35.3) | 190(27.3) | 0(0) | 0(0) |
| C9 | 50(7.2) | 204(29.3) | 252(36.2) | 185(26.5) | 6(0.9) | 0(0) |
| C10 | 77(11.0) | 324(46.5) | 160(23.0) | 127(18.2) | 9(1.3) | 0(0) |
| C11 | 31(4.4) | 108(15.5) | 233(33.4) | 186(26.7) | 129(18.5) | 10(1.4) |
| C12 | 30(4.3) | 56(8.0) | 140(20.1) | 187(26.8) | **281(40.3)** | 3(0.4) |
| C13 | 11(1.6) | 152(21.8) | 420(60.3) | 113(16.2) | 0(0) | 1(0.1) |
| C14 | 61(8.8) | 127(18.2) | 38(5.5) | 46(6.6) | **423(60.7)** | 2(0.3) |
| S1 | 12(1.7) | 27(3.9) | 242(34.7) | 394(56.5) | 22(3.2) | 0(0) |
| S2 | 28(4.0) | 80(11.5) | 240(34.4) | 339(48.6) | 10(1.4) | 0(0) |
| S4 | 56(8.0) | 194(27.8) | 189(27.1) | 251(36) | 7(1.0) | 0(0) |
| S5 | 160(23) | 386(55.4) | 118(16.9) | 31(4.4) | 2(0.3) | 0(0) |
| S6 | 82(11.8) | 110(15.8) | 225(32.3) | 274(39.3) | 0(0) | 6(0.9) |
| S7 | 7(1.0) | 44(6.3) | 261(37.4) | 384(55.1) | 0(0) | 1(0.1) |
| S8 | 97(13.9) | 215(30.8) | 181(26.0) | 204(29.3) | 0(0) | 0(0) |
| S9 | 27(3.9) | 53(7.6) | 271(38.9) | 307(44.0) | 39(5.6) | 0(0) |
| S10 | 164(23.5) | 91(13.1) | 342(49.1) | 100(14.3) | 0(0) | 0(0) |
| S11 | 120(17.2) | 213(30.6) | 157(22.5) | 204(29.3) | 0(0) | 3(0.4) |
| S12 | 182(26.1) | 306(43.9) | 91(13.1) | 118(16.9) | 0(0) | 0(0) |
| S13 | 609(87.4) | 38(5.5) | 20(2.9) | 13(1.9) | 17(2.4) | 0(0) |
| S14 | 101(14.5) | 222(31.9) | 211(30.3) | 160(23.0) | 3(0.4) | 0(0) |
| S15 | 70(10.0) | 196(28.1) | 238(34.1) | 179(25.7) | 14(2.0) | 0(0) |
| S16 | 79(11.3) | 154(22.1) | 244(35.0) | 206(29.6) | 14(2.0) | 0(0) |

(2) Inter-item and item-total correlations

Eight items (I1, I3, I4, I5, I13, C5, S5, and S13) were deleted, due to the corrected item-total correlations (CITC) <0.4 or correlation coefficient with the total score of scale<0.4 (Table 3).

Table 3. Results of Correlation coefficient (n=679)

| Item | Cronbach’s α if item deleted | Corrected item-total correlation | Correlation coefficient with the total score of scale | Item | Cronbach’s α if item deleted | Corrected item-total correlation | Correlation coefficient with the total score of scale |
| --- | --- | --- | --- | --- | --- | --- | --- |
| I1 | 0.869 | 0.339 | 0.477* | C7 | 0.933 | 0.761 | 0.654* |
| I2 | 0.866 | 0.552 | 0.558* | C8 | 0.931 | 0.779 | 0.704* |
| I3# | 0.880 | -0.104 | 0.005 | C9 | 0.934 | 0.664 | 0.612* |
| I4# | 0.879 | -0.016 | 0.028 | C10 | 0.938 | 0.620 | 0.565* |
| I5# | 0.879 | -0.047 | 0.024 | C11 | 0.931 | 0.836 | 0.823* |
| I6 | 0.858 | 0.690 | 0.632* | C12 | 0.931 | 0.768 | 0.722* |
| I7 | 0.864 | 0.642 | 0.593* | C13 | 0.933 | 0.783 | 0.701* |
| I9 | 0.861 | 0.669 | 0.623* | C14 | 0.938 | 0.617 | 0.626* |
| I10 | 0.865 | 0.563 | 0.581* | S1 | 0.904 | 0.740 | 0.702* |
| I11 | 0.858 | 0.675 | 0.659* | S2 | 0.903 | 0.770 | 0.721* |
| I12 | 0.860 | 0.662 | 0.637* | S4 | 0.914 | 0.465 | 0.450* |
| I13 | 0.877 | 0.242 | 0.109* | S5# | 0.916 | 0.282 | 0.305* |
| I14 | 0.861 | 0.707 | 0.649* | S6 | 0.905 | 0.708 | 0.675* |
| I15 | 0.859 | 0.681 | 0.656* | S7 | 0.907 | 0.741 | 0.588* |
| I16 | 0.858 | 0.705 | 0.652* | S8 | 0.901 | 0.721 | 0.714* |
| I17 | 0.865 | 0.621 | 0.603* | S9 | 0.903 | 0.724 | 0.709* |
| I18 | 0.863 | 0.668 | 0.595* | S10 | 0.909 | 0.633 | 0.646* |
| I20 | 0.863 | 0.568 | 0.616* | S11 | 0.903 | 0.680 | 0.695* |
| C1 | 0.935 | 0.664 | 0.696* | S12 | 0.909 | 0.664 | 0.620* |
| C2 | 0.938 | 0.55 | 0.586* | S13 | 0.919 | 0.091 | 0.113* |
| C3 | 0.931 | 0.746 | 0.701* | S14 | 0.900 | 0.767 | 0.759* |
| C4 | 0.930 | 0.780 | 0.755* | S15 | 0.901 | 0.784 | 0.723* |
| C5 | 0.949 | 0.274 | 0.399* | S16 | 0.899 | 0.785 | 0.760* |
| C6 | 0.932 | 0.775 | 0.723* |  |  |  |  |
| #: reverse scoring item; *: *P*<0.05 | | | | | | | |

(3) Item discrimination test

We arranged the score value of 697 patients from large to small. An upper 27% group and lower 27% group were selected to calculate whether they had statistically significant differences. As shown in Table 4, I3, I5, I13 and S13 were removed for not meeting the criteria.

Table 4. The result of item discrimination (n=679)

| **Item** | **Lower 27% group (n=189)** | **Upper 27% group (n=190)** | ***Z-value*** | ***P*-value** | **Item** | **Lower 27% group (n=189)** | **Upper 27% group (n=190)** | ***Z*-value** | ***P*-value** |
| --- | --- | --- | --- | --- | --- | --- | --- | --- | --- |
| I1 | 3.30±0.57 | 3.94±0.23 | 11.826 | <0.001 | C7 | 1.97±0.80 | 3.51±0.78 | 13.634 | <0.001 |
| I2 | 2.49±1.08 | 3.79±0.56 | 12.891 | <0.001 | C8 | 2.03±0.79 | 3.60±0.67 | 14.478 | <0.001 |
| I3* | 3.74±0.53 | 3.77±0.58 | 1.267 | 0.205 | C9 | 2.17±0.81 | 3.52±0.71 | 13.070 | <0.001 |
| I4* | 3.71±0.56 | 3.81±0.51 | 2.169 | 0.030 | C10 | 1.88±0.80 | 3.10±0.91 | 11.180 | <0.001 |
| I5* | 3.74±0.54 | 3.81±0.50 | 1.505 | 0.132 | C11 | 2.13±0.74 | 3.84±0.36 | 15.080 | <0.001 |
| I6 | 1.43±0.64 | 2.88±1.02 | 13.109 | <0.001 | C12 | 2.32±0.96 | 3.89±0.41 | 12.264 | <0.001 |
| I7 | 2.29±0.82 | 3.57±0.66 | 12.971 | <0.001 | C13 | 2.36±0.59 | 3.44±0.58 | 13.338 | <0.001 |
| I9 | 1.34±0.49 | 2.40±0.89 | 12.278 | <0.001 | C14 | 1.52±0.64 | 2.90±1.07 | 7.867 | <0.001 |
| I10 | 2.56±0.93 | 3.82±0.53 | 14.018 | <0.001 | S1 | 2.90±0.75 | 3.98±0.15 | 15.282 | <0.001 |
| I11 | 1.54±0.73 | 3.18±0.96 | 13.674 | <0.001 | S2 | 2.49±0.84 | 3.93±0.28 | 16.074 | <0.001 |
| I12 | 1.40±0.55 | 2.77±0.98 | 13.138 | <0.001 | S4 | 2.39±0.90 | 3.34±0.95 | 9.219 | <0.001 |
| I13 | 1.23±0.43 | 1.28±0.71 | -0.677 | 0.499 | S5* | 2.68±0.85 | 3.25±0.70 | 6.742 | <0.001 |
| I14 | 1.77±0.86 | 3.49±0.82 | 14.074 | <0.001 | S6 | 2.08±0.93 | 3.77±0.58 | 15.268 | <0.001 |
| I15 | 1.34±0.48 | 2.54±0.88 | 13.224 | <0.001 | S7 | 2.96±0.64 | 3.86±0.43 | 13.538 | <0.001 |
| I16 | 1.44±0.66 | 2.95±0.99 | 13.440 | <0.001 | S8 | 1.78±0.77 | 3.65±0.67 | 15.557 | <0.001 |
| I17 | 1.88±0.77 | 3.20±0.90 | 12.020 | <0.001 | S9 | 2.56±0.79 | 3.92±0.27 | 15.988 | <0.001 |
| I18 | 2.17±0.91 | 3.59±0.70 | 13.140 | <0.001 | S10 | 1.67±0.88 | 3.27±0.76 | 13.561 | <0.001 |
| I20 | 1.50±0.71 | 3.09±1.05 | 13.004 | <0.001 | S11 | 1.70±0.76 | 3.61±0.75 | 15.307 | <0.001 |
| C1 | 2.78±0.73 | 3.91±0.29 | 15.195 | <0.001 | S12 | 1.47±0.67 | 3.02±1.04 | 12.996 | <0.001 |
| C2 | 2.68±0.88 | 3.85±0.40 | 13.940 | <0.001 | S13* | 3.85±0.47 | 3.75±0.77 | -0.078 | 0.938 |
| C3 | 2.27±0.84 | 3.79±0.50 | 15.126 | <0.001 | S14 | 1.68±0.71 | 3.54±0.65 | 15.640 | <0.001 |
| C4 | 2.15±0.81 | 3.84±0.44 | 16.092 | <0.001 | S15 | 1.93±0.79 | 3.60±0.63 | 14.848 | <0.001 |
| C5 | 2.53±1.03 | 3.43±0.86 | 8.513 | <0.001 | S16 | 1.87±0.81 | 3.72±0.51 | 15.823 | <0.001 |
| C6 | 2.38±0.88 | 3.80±0.46 | 14.310 | <0.001 |  |  |  |  |  |
| *: reverse scoring item | | | | | | | | | |
